# Comprehensive analysis of lncRNAs microarray profile and mRNA–lncRNA co-expression in oncogenic HPV-positive cervical cancer cell lines

**DOI:** 10.18632/oncotarget.10232

**Published:** 2016-06-23

**Authors:** LingYun Yang, Ke Yi, HongJing Wang, YiQi Zhao, MingRong Xi

**Affiliations:** ^1^ Department of Gynecology and Obstetrics, West China Second University Hospital, Sichuan University, Chengdu 610041, China

**Keywords:** lncRNA, expression profile, coding non-coding gene co-expression, oncogenic HPV, cervical cancer

## Abstract

Long non-coding RNAs are emerging to be novel regulators in gene expression. In current study, lncRNAs microarray and lncRNA-mRNA co-expression analysis were performed to explore the alternation and function of lncRNAs in cervical cancer cells. We identified that 4750 lncRNAs (15.52%) were differentially expressed in SiHa (HPV-16 positive) (2127 up-regulated and 2623 down-regulated) compared with C-33A (HPV negative), while 5026 lncRNAs (16.43%) were differentially expressed in HeLa (HPV-18 positive) (2218 up-regulated and 2808 down-regulated) respectively. There were 5008 mRNAs differentially expressed in SiHa and 4993 in HeLa, which were all cataloged by GO terms and KEGG pathway. With the help of mRNA-lncRNA co-expression network, we found that ENST00000503812 was significantly negative correlated with RAD51B and IL-28A expression in SiHa, while ENST00000420168, ENST00000564977 and TCONS_00010232 had significant correlation with FOXQ1 and CASP3 expression in HeLa. Up-regulation of ENST00000503812 may inhibit RAD51B and IL-28A expression and result in deficiency of DNA repair pathway and immune responses in HPV-16 positive cervical cancer cell. Up-regulation of ENST00000420168, ENST00000564977 and down-regulation of TCONS_00010232 might stimulate FOXQ1 expression and suppress CASP3 expression in HPV-18 positive cervical cancer cell, which lead to HPV-induced proliferation and deficiency in apoptosis. These results indicate that changes of lncRNAs and related mRNAs might impact on several cellular pathways and involve in HPV-induced proliferation, which enriches our understanding of lncRNAs and coding transcripts anticipated in HPV oncogenesis of cervical cancer.

## INTRODUCTION

Widespread uptake of the Papanicolaou test for cervical cancer screening and advanced medical treatments have reduced the incidence by 40%-50% and the mortality by 60% in many developed countries [1]. However, carcinoma of the uterine cervix still ranks the fourth most common cancer in women worldwide with 85% of cases occurring in developing countries, where cervical cancer is a leading cause of cancer death in women [2]. The morbidity in China is still considerable with almost one-third of the total number worldwide, which remains one of the most important issues in women's health care [3].

It is proved that cervical cancer is associated with persistent oncogenic human papillomavirus (HPV) infections causing atypical hyperplasia. This is regarded as the most crucial factor contributing to the development of this deadly disease [4]. Oncogenic HPVs, also known as high-risk HPVs including HPV-16, HPV-18 and HPV-53, can be detected in up to 99.7% patients with cervical cancer [5]. Among all oncogenic HPVs identified, HPV-16 and HPV-18 are the two most common types contributing to 70% of invasive cervical cancer [6]. HPV DNA is found integrated into the host chromosomes in most cases of cervical carcinoma, increasing the expression of E6 and E7 [7, 8], destabilizing two major cellular tumor suppressor proteins p53 and retinoblastoma protein (pRB) and blocking cell cycle exit during differentiation. Consistently elevated expression of E6 and E7 can lead to immortalization and transformation in oncogenic HPV-positive cells [9, 10].

A new class of transcripts, long non-coding RNAs (lncRNAs), has been recently identified to be pervasively transcribed in eukaryotic genome [11, 12]. LncRNA, ranging from approximately 200nt to over 100kb in length, is one of functional non-coding RNA molecules. It is capable of regulating gene expression during cellular differentiation [13, 14], governing a wide-repertoire of molecular biological and genetic processes, including transcription [15], translation [16], splicing [17], imprinting [18], differentiation [19], chromatin modification [20], chromatin structure [21], cell cycle control [22], cellular structure [23] and stem cells regulation [24]. Moreover, increasing evidences suggest that altered expression of lncRNAs could be associated with genesis and progression of many human diseases, even malignant cancers [25–27]. However, few studies have evaluated the alternation and function of lncRNAs in oncogenic HPV-positive cervical cancer cells.

In current study, we performed a high-throughput analysis to detect the global lncRNAs and mRNAs expression changes in oncogenic HPV-positive cervical cancer cell lines. Additionally, we constructed a co-expression network of lncRNAs and coding gene transcripts to predict the potential biological function of differentially expressed lncRNAs. Our study might help to understand the interplay between lncRNAs and coding genes anticipated in oncogenic HPV proliferation, and lncRNAs may be the missing links of well-known oncogenic and tumor suppressor networks.

## RESULTS

### Overview of lncRNAs expression

**Figure 1:**
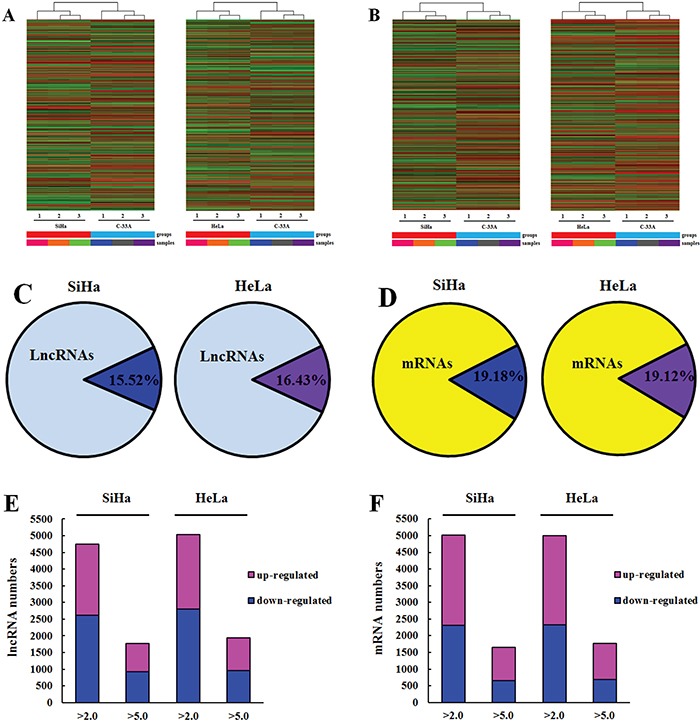
Oncogenic HPV infection regulates the expression of host lncRNAs and mRNA in cervical cancer cells **A, B.** Hierarchical clustering of lncRNAs (A) and mRNAs (B) differently expressed in SiHa and HeLa comparing with C-33A. “Red” indicates high relative expression, and “blue” indicates low relative expression. **C, D.** Ratio of differentially expressed lncRNAs (C) and mRNAs (D) in SiHa and HeLa compared with C-33A. Lighter colors refer to the total number of transcripts, and darker colors refer to the number of differentially expressed lncRNAs and mRNAs. **E, F.** Column chart of differentially expressed lncRNAs (E) and mRNAs (F) in SiHa and HeLa. Bar plots show the number of increased transcripts (purple) or decreased transcripts (blue) at different fold change cut-off values.

In order to determine differentially expressed lncRNAs in oncogenic HPV-positive cervical cancer, we performed a microarray analysis among three kinds of cervical cancer cell lines. A total of 30586 lncRNAs were detected. Differentially expressed lncRNAs were selected only when they were altered by fold change cut-off 2.0. We identified that 4750 lncRNAs (15.52%) were differentially expressed in SiHa (HPV-16 positive) (2127 up-regulated and 2623 down-regulated) compared with C-33A (HPV negative), while 5026 lncRNAs (16.43%) were differentially expressed in HeLa (HPV-18 positive) (2218 up-regulated and 2808 down-regulated) respectively (P<0.05 and FDR<0.05) (Figure 1A, 1C, 1E; Table 1). 1780 lncRNAs exhibited a high fold change (over 5-fold) in HPV-16 positive cervical cancer cells (845 increased and 935 decreased). Moreover, 1835 lncRNAs had an over 5 fold change in HPV-18 positive cervical cancer cells (960 increased and 975 decreased) (Figure 1E, Table 1).

**Table 1:** Numbers of differentially expressed lncRNAs in SiHa and HeLa compared with C-33A

LncRNAs	SiHa		HeLa	
	Fold change>2.0	Fold change>5.0	Fold change>2.0	Fold change>5.0
Up-regulated	2127 (44.8%)	845 (47.5%)	2218 (44.1%)	975 (53.1%)
Down-regulated	2623 (55.2%)	935 (52.5%)	2808 (55.9%)	960 (46.9%)
Total	4750	1780	5026	1835

### Overview of mRNAs profiles

Hierarchical clustering analysis was applied to group mRNAs based on their expression level (Figure 1B). From the microarray data, a total of 26109 coding transcripts were detected, 5008 out of which, approximately 19.18% were differentially expressed in SiHa. Among the 5008 mRNAs, 2698 mRNA were increased and 2310 were decreased (P<0.05 and FDR<0.05). In HeLa, 4993 out of 26109 (19.12%) mRNAs showed aberrant expression, among which 2667 mRNA were increased and 2326 mRNA were decreased (P<0.05 and FDR<0.05) (Figure 1D, 1F; Table 2). There were 1657 mRNAs exhibiting a high fold change (over 5-fold) in SiHa (1022 mRNA up-regulated and 655 mRNA down-regulated), while 1773 mRNA showing over 5-fold change in HeLa (1008 mRNA up-regulated and 685 mRNA down-regulated) (Figure 1F; Table 2).

**Table 2:** Numbers of differentially expressed mRNAs in SiHa and HeLa compared with C-33A

mRNAs	SiHa		HeLa	
	Fold change>2.0	Fold change>5.0	Fold change>2.0	Fold change>5.0
Up-regulated	2698 (53.9%)	1002 (60.5%)	2667 (53.4%)	1088 (56.9%)
Down-regulated	2310 (46.1%)	655 (39.5%)	2326 (46.6%)	685 (43.1%)
Total	5008	1657	4993	1773

### Subgroups of differentially expressed lncRNAs

**Figure 2:**
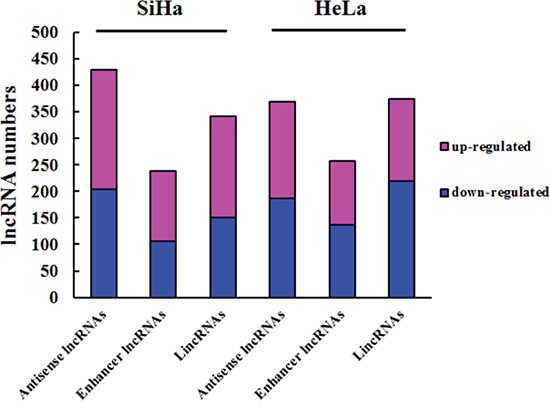
Subgroups of differentially expressed lncRNAs Column chart show three kinds of lncRNAs in HeLa and SiHa compared with C-33A. Bar plots indicate the number of increased transcripts (purple) or decreased lncRNAs (blue).

**Figure 3:**
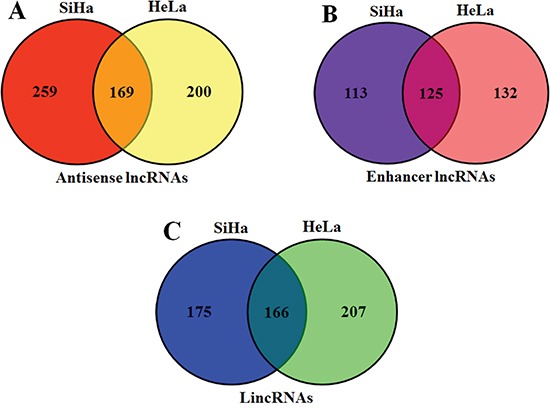
Venn diagrams of three kinds of lncRNAs There were 169 antisense lncRNAs **A.,** 125 enhancer lncRNAs **B.** and 166 lincRNAs **C.** were synchronously up or down regulated in both SiHa and HeLa.

We continuously adapted specific probes for lncRNAs to classify and subgroup three kinds of lncRNA: antisense lncRNAs, enhancer lncRNAs and intergenic lncRNAs (lincRNAs). The profiling data suggested that 428 antisense lncRNAs, 238 enhancer lncRNAs and 341 lincRNAs could be detected differentially expressed in SiHa comparing with C-33A, while 369 antisense lncRNAs, 257 enhancer lncRNAs and 373 lincRNAs in HeLa (P<0.05 and FDR<0.05) (Figure 2). Furthermore, we identified 169 antisense lncRNAs, 125 enhancer lncRNAs and 166 lincRNAs, which were synchronously up or down regulated in both SiHa and HeLa using Venn diagrams [28] (Figure 3).

### Gene ontology and KEGG pathway analysis

**Figure 4:**
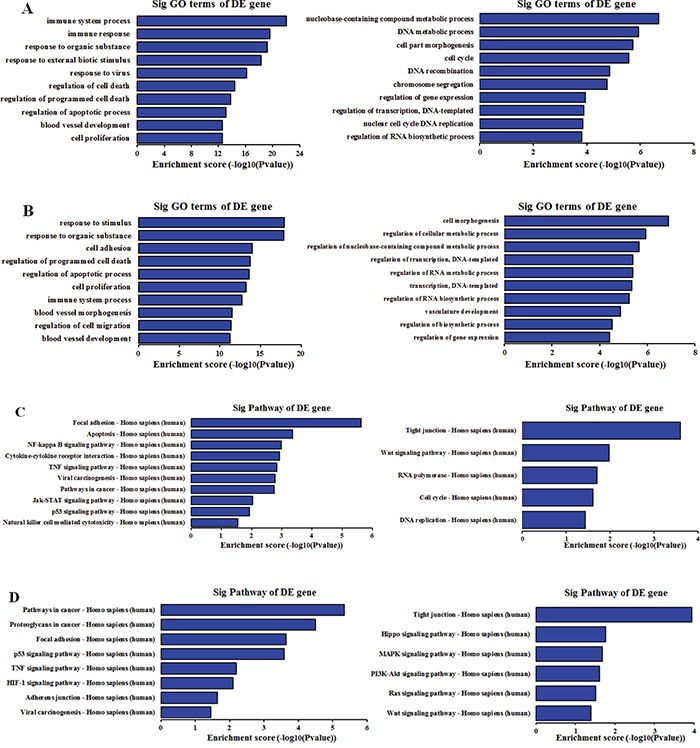
GO and KEGG pathway analysis of differentially expressed mRNAs in SiHa and HeLa as regards to biological processes **A.** Top 10 GO terms enriched among up-regulated and down-regulated mRNAs in SiHa. **B.** Top 10 GO terms enriched among up-regulated and down-regulated mRNAs in HeLa. **C.** The most enriched pathway among among up-regulated and down-regulated mRNAs in SiHa. **D.** The highest enriched pathway among among up-regulated and down-regulated mRNAs in HeLa. The P-value indicates the significance of the correlation between the pathway and HPV infection.

Gene ontology (GO) analysis for all of the differentially expressed mRNAs was performed to identify the function of coding transcripts. It is reported that lncRNAs could regulate the expression of adjacent or overlapping coding genes [29]. As a result, the function of these coding genes might provide insight into these differentially expressed lncRNAs. As regards to biological processes, the most enriched GO terms associated with up-regulated mRNA were the immune response, response to organic substance and response to virus among others in SiHa, and the response to stimulus, response to organic substance, cell adhesion among others in HeLa. In addition, the highest enriched GO terms targeted by down-regulated mRNAs included DNA metabolic process, cell morphogenesis and cell cycle among others in SiHa, and cell morphogenesis, regulation of cellular metabolic process and regulation of transcription among others in HeLa. Interestingly, our results showed that the up-regulated mRNAs related to biological processes were specific to response to organic substance, cell proliferation, blood vessel development, regulation of programmed cell death and apoptosis in both SiHa and HeLa, while the down-regulated mRNA were largely associated with cell morphogenesis, DNA metabolic, DNA recombination and regulation of transcription (Figure 4A, 4B).

Kyoto Encyclopedia of Genes and Genomes (KEGG) pathway analysis was applied to make a deep understanding between the differentially expressed mRNAs and cell pathways. Our results indicated that 26 pathways were significantly enriched among the up-regulated mRNAs and 5 pathways were significantly enriched among the down-regulated mRNAs in SiHa. In addition, 8 pathways corresponded to up-regulated transcripts and 6 pathways to down-regulated transcripts. Importantly, viral carcinogenesis, focal adhesion, p53 signaling pathway and TNF signaling pathway were associated with up-regulated mRNAs in both SiHa and HeLa, whereas tight junction, Wnt signaling pathway, cell cycle were significantly related to down-regulated mRNA (Figure 4C, 4D).

### Construction of the coding non-coding gene co-expression network

**Figure 5:**
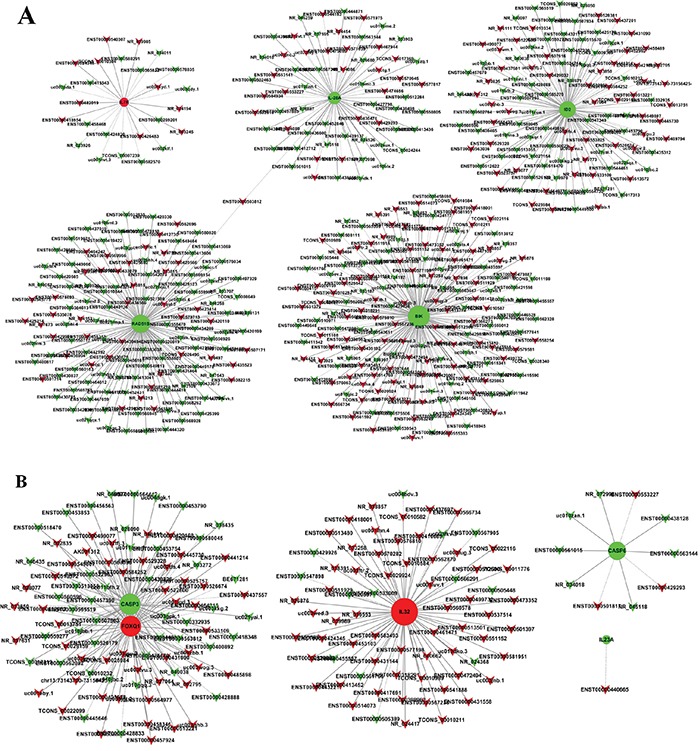
Coding non-coding gene co-expression networks in SiHa A. and HeLa B “Triangle node” represents as the lncRNAs and “round node” represents as the mRNA. “Red” indicates up-regulated genes and “green” indicates down-regulated genes. “Solid line” indicates a positive correlation; “dashed line” indicates a negative correlation.

The coding non-coding gene (CNC) co-expression network was constructed based on the correlation analysis. LncRNAs and mRNAs with PCC≥0.99 were selected to draw the network. Respectively, 5 differently expressed coding genes were selected from each SiHa and HeLa. These coding genes are involved in multiple biological processes including DNA repair, cell cycle, cell death, apoptosis and immune response according to the GO and KEGG pathway analysis results. The CNC network indicated that one mRNA could correlate with hundreds of lncRNAs. Among this co-expression network, there was only one lncRNA (ENST00000503812) negative correlated with RAD51 paralog B (RAD51B) and Interleukin 28A (IL-28A) in SiHa (Figure 5A). Consequently, we selected ENST00000503812 for further validation. While in HeLa, there were 153 differentially expressed lncRNAs correlated with Forkhead box Q1 (FOXQ1) and Caspase-3 (CASP3) expression in HeLa. Among the correlated lncRNAs, we selected one antisense lncRNA (ENST00000420168) and two lincRNAs (ENST00000564977, TCONS_00010232) for further validation. ENST00000420168 and ENST00000564977 showed positive correlation with FOXQ1 expression and negative correlation with CASP3 expression in HeLa, while TCONS_00010232 was negative correlated with FOXQ1 and positive correlated with CASP3 (Figure 5B).

### Real-time qPCR and western blot validation

**Figure 6:**
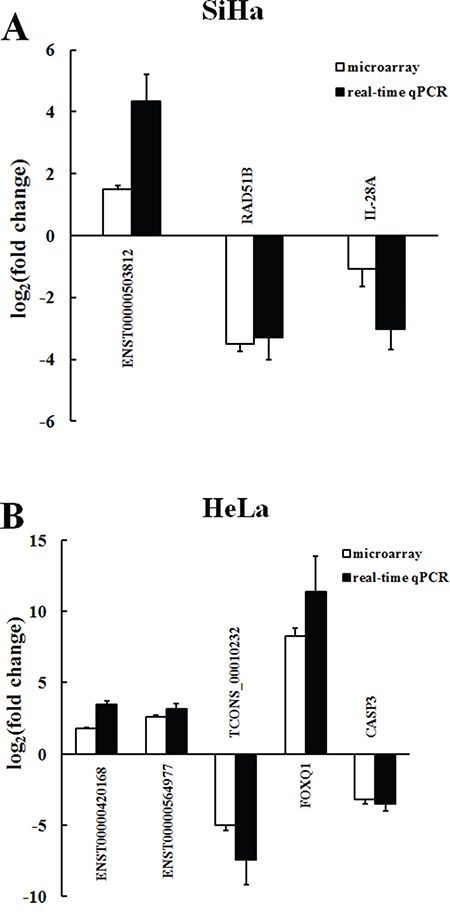
Comparison between microarray and real-time qPCR for differentially expressed lncRNAs and mRNAs in SiHa A. and HeLa B The heights of the columns represent the log-transformed mean fold change in expression compared with C-33A. The bars represent standard errors. The real-time qPCR results were consistent with the microarray data (P<0.001).

**Figure 7:**
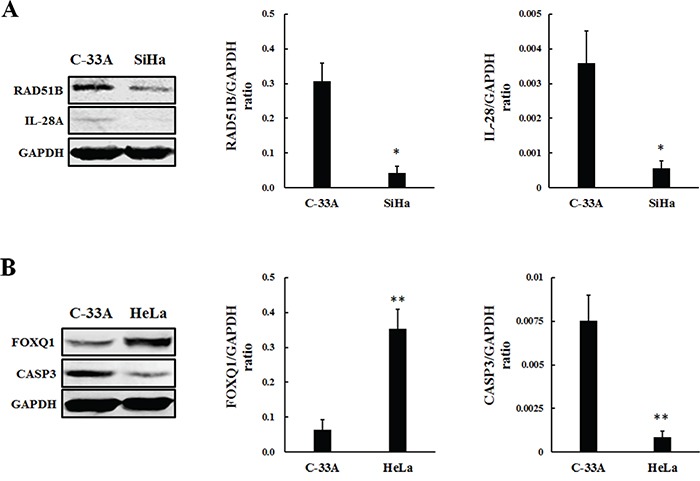
Protein level changes of lncRNAs related coding genes in SiHa A. and HeLa B Ratios of coding gene signal divided by GAPDH signal were calculated. (A) The expression of RAD51B and IL-28A were down-regulated in SiHa compared with C-33A (*P<0.01). (B) The expression of FOXQ1 increased in HeLa, whereas CASP3 decreased compared with C-33A (**P<0.01).

Real-time qPCR (RT-qPCR) was performed for further validation of selected differentially expressed lncRNAs and mRNAs from the CNC co-expression network. High values of relative threshold cycles indicate the low quantity, which were normalized by internal control GAPDH expression. The RT-qPCR results were correlated well with the microarray data. ENST00000503812 was up-regulated, while RAD51B and IL-28A were down-regulated in SiHa compared with C-33A (P<0.0001) (Figure 6A; Table 3). Additionally, the expression of ENST00000420168, ENST00000564977 and FOXQ1 were up-regulated in HeLa, whereas TCONS_00010232 and CASP3 were down-regulated compared with C-33A (P<0.0001) (Figure 6B; Table 4). Protein level changes of lncRNAs related coding genes were detected by western blot. Ratios of coding gene signal divided by GAPDH signal were calculated. We found that expression of RAD51B and IL-28A were down-regulated in SiHa compared with C-33A (P<0.01) (Figure 7A). In HeLa, expression of FOXQ1 increased, whereas CASP3 decreased compared with C-33A (P<0.01) (Figure 7B). These results suggested that the expression changes at protein level were consistent with microarray and RT-qPCR data.

**Table 3:** Validation of microarray data by real-time qPCR in SiHa

SiHa vs C-33A		LncRNA	mRNA	
		ENST00000503812	RAD51B	IL-28A
Microarray	Regulation	up	down	down
	fold change^*^	2.8043	11.2705	2.0913
Real-time qPCR	Regulation	up	down	down
	fold change^**^	20.1349	9.7160	8.0909

* P<0.001;

** P<0.001

**Table 4:** Validation of microarray data by real-time qPCR in HeLa

HeLa vs C-33A		LncRNA			mRNA	
		ENST00000420168	ENST00000564977	TCONS_00010232	FOXQ1	CASP3
Microarray	Regulation	up	up	down	up	down
	fold change^*^	1.7657	2.5949	4.9937	8.2470	9.3561
Real-time qPCR	Regulation	up	up	down	up	down
	fold change^**^	3.4570	3.1723	7.4789	11.3909	11.4285

* P<0.001;

** P<0.001

## DISCUSSION

LncRNAs are emerging to be functional regulators in various biological processes impacting cellular development, differentiation and metabolism [12]. These novel characterized non-coding transcripts have recently become the hotspot of attention due to their complex and extensive roles in cancer development and progression [26]. With the advent of new technologies achieving unprecedented depths in RNA sequencing, tens of thousands of lncRNAs have been identified across the mammalian genome. However, there is a lack of comprehensive databases that provide a resource for experimentally verified lncRNA function. Nowadays, integration of lncRNAs and coding genes expression profiles becomes one of the most universal ways to study lncRNA functions in different biological processes and cancers. In this study, we selected three kinds of cervical cancer cell lines including SiHa (contain an integrated HPV-16 genome), HeLa (contain an integrated HPV-18 genome) and C-33A (HPV DNA and RNA negative), in which host lncRNAs and mRNAs were comprehensively investigated.

The microarray data showed that thousands of host lncRNAs had differential expression in oncogenic HPV-positive cervical cancer cells (4750 in SiHa and 5026 in HeLa). According to the relationship between lncRNAs and their associated protein-coding genes, lncRNAs can be characterized as antisense, enhancer, intergenic, bidirectional and intronic lncRNAs. Based on these specialized classifications, we performed the subgroup analysis among the differentially expressed lncRNAs and identified hundreds of differentially expressed antisense, enhancer and intergenic lncRNAs. Particularly, antisense lncRNAs are transcribed from the antisense strand and can regulate a wide variety of biological processes via diverse transcriptional and post-transcriptional gene regulatory mechanisms [30], and enhancer lncRNAs are transcribed from the DNA sequence of enhancer regions which actively facilitate transcription of protein-coding genes [31]. Moreover, there are a large number of non-coding regions interspersed between coding regions [32], where intergenic lncRNAs (lincRNAs) are transcribed from. LincRNAs perform important functions in many cellular processes, from embryonic stem cell pluripotency to cell proliferation and cancer progression [33, 34]. The changes of mRNAs were also investigated from microarray data. 5008 mRNAs were found differentially expressed in SiHa and 4993 in HeLa. In order to reveal the biological function, we adapted GO and KEGG analysis to provide a deep insight of these mRNAs involved in some biological processes. GO analysis showed that altered coding genes in HPV-positive cervical cancer cells were mainly associated with immune response, response to virus, regulation of apoptosis, regulation of transcription and DNA recombination. KEGG pathway analysis also revealed that viral carcinogenesis, apoptosis, cell cycle, DNA replication and p53 signaling pathway among others were the most enriched pathways targeted by differentially expressed coding genes. With the help of CNC network, we found that ENST00000503812 was negative correlated with RAD51B and IL-28A in SiHa, while ENST00000420168 and ENST00000564977 were positive correlated with the expression FOXQ1 and CASP3 in HeLa, and TCONS_00010232 had a negative correlation. The validation of lncRNAs and associated mRNAs expression was consistent with the microarray data at both RNA and protein levels. Consequently, our findings showed that the integration of oncogenic HPV DNA can alter most kinds of host lncRNAs expression and exert effects on the development and progress of cervical cancer via regulating related mRNAs expression.

Combination of lncRNAs and coding genes expression profiles helps us to have a better understanding of biological functions in HPV-positive cervical cancer cells. Integration of oncogenic HPV DNA into the host genome is proved to via enhanced expression of viral oncoproteins, alteration of critical cellular genes, and changes of transcription in cervical cancer. Coding genes alternation may lead to loss of function of tumor suppressor genes, overexpressed oncogene expression, loss of function of DNA repair genes, or other vital cellular functions [35]. RAD51B is a component of the DNA double-strand break repair pathway, and loss of this gene may promote genomic instability [36]. It has been noted that RAD51B is disrupted by HPV integration in multiple cervical cancer samples [37], which is consistent with our results. Meanwhile, IL-28A, also known as interferon lambda 2 (IFN-L2), plays an active role in the adaptive immune response and leads to antigen-specific interferon gamma (IFN-γ) release as well as inducing FoxP3+ regulatory T cells [38, 39]. Recently, it is reported that IL-28 has potential anti-tumor effect against human lung cancer and also associates with hepatitis C virus infection [40, 41]. Moreover, we demonstrated that lncRNA ENST00000503812 was significantly negative correlated with RAD51B and IL-28A expression in SiHa. Up-regulation of ENST00000503812 may inhibit RAD51B and IL-28A expression and result in deficiency of DNA repair pathway and immune responses in HPV-16 positive cervical cancer cell. Oncogene FOXQ1 is overexpressed in a series of tumors, such as hepatocellular carcinoma [42], breast cancer [43], and colorectal cancer [44] and becomes a potential bio-marker in prognosis. CASP3 is well recognized as an apoptosis-related gene and induces efficient cell death [45]. Our results showed that ENST00000420168, ENST00000564977 and TCONS_00010232 had significant correlation with FOXQ1 and CASP3 expression in HeLa. Up-regulation of ENST00000420168, ENST00000564977 and down-regulation of TCONS_00010232 might stimulate FOXQ1 expression and suppress CASP3 expression in HPV-18 positive cervical cancer cell, which lead to HPV-induced proliferation and deficiency in apoptosis. These results indicate that changes of lncRNAs and related mRNAs might impact on several cellular pathways and involve in HPV-induced proliferation.

In conclusion, our study demonstrated that integration of oncogenic HPV DNA into host genome could alter the expression profiles of lncRNAs in cervical cancer cells. Co-expression network revealed the correlation between lncRNAs and mRNAs, and certain lncRNAs can lead to differentially expression of their target protein-coding genes including several crucial regulators of DNA repairing, cell cycle, proliferation and apoptosis in HPV-positive cervical cancer cell lines. These findings could help enrich our understanding of lncRNAs and coding transcripts anticipated in HPV oncogenesis of cervical cancer.

## MATERIALS AND METHODS

### Cervical cancer cell lines

Cervical cancer cell lines SiHa (grade II squamous cell carcinoma, containing an integrated HPV-16 genome), HeLa (adenocarcinoma, containing an integrated HPV-18 genome) and C-33A (carcinoma, negative for HPV DNA and RNA) were all grown in Dulbecco's Modified Eagle Medium (DMEM, Gibco) supplemented with 10% fetal bovine serum (FBS, Gibco). All cell lines were cultured in a humidified incubator with 5% CO_2_ at 37°C. Growth and morphology of each cell line were observed and monitored every three days. All cell lines were obtained from China Center for Type Culture Collection, Wuhan.

### RNA isolation

Total RNA was isolated using TRIzol reagent (Invitrogen) according to manufacturer's protocol. The concentration and quality of total RNA were measured by NanoDrop ND-1000 spectrophotometry (Thermo Scientific) and standard denaturing agarose gel electrophoresis.

### RNA labeling and array hybridization

Sample labeling and array hybridization were performed according to the Agilent One-Color Microarray-Based Gene Expression Analysis protocol (Agilent Technology) with minor modifications. Briefly, mRNA was purified from total RNA after removal of rRNA (mRNA-ONLY™ Eukaryotic mRNA Isolation Kit, Epicentre). Then, each sample was amplified and transcribed into fluorescent cRNA along the entire length of the transcripts without 3′ bias utilizing a random priming method (Arraystar Flash RNA Labeling Kit, Arraystar). The labeled cRNAs were purified by RNeasy Mini Kit (Qiagen). The concentration and specific activity of the labeled cRNAs were measured by NanoDrop ND-1000 spectrophotometry (Thermo Scientific). 1μg of each labeled cRNA was fragmented by adding 5μl 10× blocking agent and 1μl of 25× fragmentation buffer, then heated the mixture at 60°C for 30 min, finally 25μl 2× GE Hybridization buffer was added to dilute the labeled cRNA. 50μl of hybridization solution was dispensed into the gasket slide and assembled to the lncRNA expression microarray slide. The slides were incubated for 17 hours at 65°C in an Agilent Hybridization Oven. The hybridized arrays were washed, fixed and scanned using the Agilent Microarray Scanner.

### Microarray data analysis

Agilent Feature Extraction software (version 11.0.1.1) was used to analyze acquired array data. Quantile normalization and subsequent data processing were performed with using the GeneSpring GX v12.1 software package (Agilent Technologies). We determined 30,586 LncRNAs and 26,109 coding transcripts from authoritative data sources including Gencode, National Center for Biotechnology Information (NCBI) RefSeq, University of California Santa Cruz (UCSC) known gene and lncRNAs from literatures and Ultra Conserved Regions (UCRs). Differentially expressed lncRNAs and mRNAs with statistical significance between the two groups were identified through P-value, false discovery rate (FDR) and fold change filtering. Hierarchical Clustering and combined analysis were performed using homemade scripts.

### Gene ontology and KEGG pathway analysis

Gene ontology (GO) is a major bioinformatics to describe gene and gene product attributes across all species (http://geneontology.org/). It provides an ontology of defined terms representing gene product properties, which covers three domains: cellular component, molecular function and biological process [46, 47]. Two-side Fisher's exact test was applied to detect overlap between the differentially expressed list and the GO annotation list which was greater than that expected by chance. FDR was calculated to correct the P-value. Enrichment scores were calculated among differentially expressed genes, and as the enrichment increases, the corresponding function is more specific. We also adopted KEGG pathway (http://www.genome.jp/kegg/) to map differentially expressed genes. The enrichment and statistics calculation were similar to the GO analysis.

### Coding non-coding gene co-expression network

The coding non-coding gene co-expression network was constructed to explore the relationship between lncRNAs and mRNAs. The procedures included that: (I) to preprocess data, the median value of the same coding gene with different transcripts was calculated, which could represents as the gene expression values; (II) screened data and collected the subset of data according to the lists of differentially expressed lncRNAs and mRNAs; (III) Pearson correlation coefficient (PCC) was calculated between lncRNAs and coding genes. (IV) PCC≥0.99 was considered to be meaningful correlation. Cytoscape (v3.3.0) was used to illustrate the co-expression network.

### Real-time quantitative PCR analysis

Verification of four lncRNAs (ENST00000503812, ENST00000420168, ENST00000564977 and TCONS_00010232) and related mRNAs (RAD51B, IL-28A, FOXQ1 and CASP3) expression were performed by RT-qPCR. Total RNA of each cell line was extracted using Trizol reagent (Invitrogen) and assessed by agarose gel electrophoresis and spectrophotometer. cDNA was generated using SuperScript™ III Reverse Transcriptase (Invitrogen). RT-qPCR was performed in a 10μL reaction volume containing 2μL cDNA template, 5μL 2× Master Mix (Arraystar), 0.5μL 10μM forward primer, 0.5μL 10μM reverse primer and 2μL RNase-free H_2_O on ViiA 7 Real-time PCR System (Applied Biosystems) using the following protocol: 95°C for 10min, followed by 40 cycles of 95°C for 10s, 60°C for 60s and 95°C for 15s. Each sample was run in triplicate. The relative expression was calculated using 2^−ΔΔCt^ method.

### Western blot analysis

Validation of related mRNAs (RAD51B, IL-28A, FOXQ1 and CASP3) expression at protein level was performed by western blot. After 48h, the cell culture supernatants (containing IL-28A) were collected and concentrated by ultrafiltration and filtered through a 0.45mm membrane. Other proteins were extracted with RIPA buffer (Thermo Scientific) supplemented with protease inhibitor cocktail (Sigma-Aldrich). Equal amount of proteins was resolved 10% SDS-PAGE, transferred to PVDF membrane (Bio-Rad). The membranes were blocked with 5% nonfat dry milk in TBST buffer (25mM Tris-HCl, 125mM NaCl, 0.1% Tween 20) for 1 hour and probed with primary antibodies overnight. After washing 3 times with TBST buffer, the membranes were incubated with IRDye 800CW- or IRDye 680RD-conjugated secondary antibodies (LI-COR Biosciences) for 1 hour. The results were visualized using an Odyssey Infrared Imager (LICOR Biosciences). For loading control, the membranes were stripped and probed for glyceraldehyde-3-phosphate dehydrogenase (GAPDH). The antibodies used are as follows: rabbit anti-RAD51B, rabbit anti-IL-28A and mouse anti-FOXQ1 antibodies were obtained from Abcam (Cambridge, MA, USA), rabbit anti-CASP3 antibody was obtained from Cell Signaling Technology (Danvers, MA, USA).

### Statistical analysis

Differences among groups were evaluated by two-tailed Student's t-test by using SPSS Statistics v20.0 software (IBM). P<0.05 was considered to be statistically significant.
